# Linear and Nonlinear Dynamic Behavior of Polymer Micellar Assemblies Connected by Metallo-Supramolecular Interactions

**DOI:** 10.3390/polym11101532

**Published:** 2019-09-20

**Authors:** Zhi-Chao Yan, Florian J. Stadler, Pierre Guillet, Clément Mugemana, Charles-André Fustin, Jean-François Gohy, Christian Bailly

**Affiliations:** 1 Shenzhen Key Laboratory of Polymer Science and Technology, Guangdong Research Center for Interfacial Engineering of Functional Materials, College of Materials Science and Engineering, Shenzhen University, Shenzhen 518060, China; yanzhch@szu.edu.cn; 2 Institute of Condensed Matter and Nanosciences (IMCN), Bio and Soft Matter Division (BSMA), Université Catholique de Louvain, Place Pasteur 1, B-1348 Louvain-la-Neuve, Belgium; pierre.guillet@univ-avignon.fr (P.G.); clement.mugemana@list.lu (C.M.); charles-andre.fustin@uclouvain.be (C.-A.F.); jean-francois.gohy@uclouvain.be (J.-F.G.); 3 Equipe Chimie Bioorganique et Systèmes Amphiphiles, Institut des Biomolécules Max Mousseron (UMR 5247 UM-CNRS-ENSCM) & Avignon University, 301 rue Baruch de Spinoza, 84916 Avignon CEDEX 9, France; 4 Luxembourg Institute of Science and Technology (LIST), 5 Avenue des Hauts-Fourneaux, 4362 Esch-sur-Alzette, Luxembourg; 5 Institute of Condensed Matter and Nanosciences (IMCN), Bio and Soft Matter Division (BSMA), Université Catholique de Louvain, Place Croix du Sud 1, B-1348 Louvain-la-Neuve, Belgium; christian.bailly@uclouvain.be

**Keywords:** associative polymer colloids, micellar assemblies, rheology

## Abstract

The linear and nonlinear rheology of associative colloidal polymer assemblies with metallo-supramolecular interactions is herein studied. Polystyrene-*b*-poly(*tert*-butylacrylate) with a terpyridine ligand at the end of the acrylate block is self-assembled into micelles in ethanol, a selective solvent for the latter block, and supramolecularly connected by complexation to divalent metal ions. The dependence of the system elasticity on polymer concentration can be semi-quantitatively understood by a geometrical packing model. For strongly associated (Ni^2+^, Fe^2+^) and sufficiently concentrated systems (15 w/v%), any given ligand end-group has a virtually 100% probability of being located in an overlapping hairy region between two micelles. By assuming a 50% probability of intermicellar crosslinks being formed, an excellent prediction of the plateau modulus was achieved and compared with the experimental results. For strongly associated but somewhat more dilute systems (12 w/v%) that still have significant overlap between hairy regions, the experimental modulus was lower than the predicted value, as the effective number of crosslinkers was further reduced along with possible density heterogeneities. The reversible destruction of the network by shear forces can be observed from the strain dependence of the storage and loss moduli. The storage moduli of the Ni^2+^ and Zn^2+^ systems at a lower concentration (12 w/v%) showed a rarely observed feature (i.e., a peak at the transition from linear to nonlinear regime). This peak disappeared at a higher concentration (15 w/v%). This behavior can be rationalized based on concentration-dependent network stretchability.

## 1. Introduction

Supramolecular self-assembly is one of the most fascinating topics in soft matter. It gives synthetic materials some of the merits of biological systems, including self-healing properties, response to external stimuli, and the ability to reversibly modify stiffness as a reaction to external stress levels [1,2,3,4,5,6,7,8,9,10,11,12,13,14,15,16,17,18]. Supramolecular self-assembly strategy has been shown to work excellently for constructing dynamically bonded gels [19,20]. In particular, supramolecular micellar gels can be created with somewhat slower dynamic behavior, according to a binary self-assembly strategy that combines both phase-separation-induced block aggregation and the bonding from supramolecular intermicellar interactions [7]. As regards micellar gels made by block copolymers, a kinetically controlled and even reversible self-assembly is possible, making these gels good candidates for self-repairing and injectable soft materials.

So far, the research of supramolecular micellar gels is still mainly focused on chemically creating new functionality and designing new structures. The material’s physical properties have not yet been thoroughly evaluated, especially the relationship between rheological behavior and structural information like micelle size, intermicellar distance, relative volume of core and corona, and strength of supramolecular interactions. In an earlier study, the structure of a wormlike micelle supramolecularly bridged by telechelic polymers was explored by Ligoure et al. [6]. A similar approach was also conducted by Lodge et al. [21,22], who found evidence for establishing crosslinks between wormlike micelles by a poly(ethylene oxide) with alkyl stickers installed at the chain ends. However, their samples were mixtures of micelles and linear chains, which cannot answer the question in pure micellar gels of how supramolecular networks are formed simply by intermicellar bonding among arms in coronae. 

**Scheme 1:**
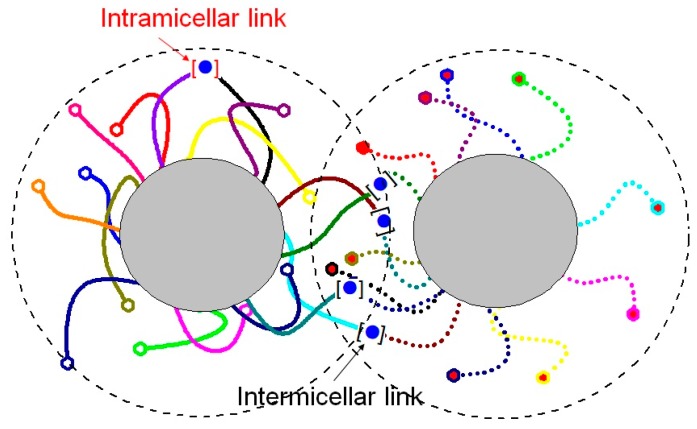
Cartoon for connection formed by self-assembly of PS-*b*-PtBA with a terpyridine ligand. The core and coronae are PS and PtBA, respectively. ● Represents the metal ion.

Among the different non-covalent interactions forming supramolecular bonds, the metal–ligand bond is particularly interesting because of its great directionality, broad selection of ligands, and easily tuned interaction strength that can be selected by choosing the appropriate metal ion. Guillet et al. [7,15] combined colloidal self-assembly and metallo-supramolecular interactions by exploring the behavior of polystyrene-*b*-poly(*tert*-butylacrylate) (PS-*b*-PtBA) with a terpyridine ligand at the end of the acrylate block, which is self-assembled in ethanolic solution. The addition of divalent ions such as Zn^2^, Ni^2+^, and Fe^2+^ yield flower-like micelles in diluted solutions and temporary networks in concentrated and semi-concentrated systems. The connections formed by such micelles are shown in Scheme 1. The rheological behavior of such gels can be extensively tuned by choice of metal ion. Besides metal–terpyridine coordination, we have also reported interactions between dopamine and metal ions in thermosensitive copolymers for self-assembly into switchable polymer gels. This process depends on a variety of factors, including pH [23,24], temperature [23], solvent type [19,24,25], ion type [26], and nanoparticles [27,28]. In most studies, the rheological properties have been restricted to a few test setups. In these preliminary studies, a frequency sweep was often carried out to prove gelation at an almost constant phase angle δ (or a constant slope in storage and loss modulus) [20,29], while the absence of such a constant slope has indicated that the material is sufficiently elastic (as in the case of a typical entangled polymer) [30]. However, a relationship between these rheological results (e.g., the plateau modulus and structural and connecting situations of micelles) have not been theoretically established. In addition, the nonlinear rheological behavior of micellar gels has not been thoroughly explored.

This paper expands on an understanding of the concentrated systems. It focuses on the surprising finding that even small concentration differences can completely change rheological characteristics. Based on simple geometric considerations as well as structural and chemical information about this system, we first calculate a key structural parameter—the intermicellar linking fraction—then consider implications on gelling behavior. Predictions are compared with linear rheological measurements for two concentrations (12 and 15 w/v%) and three ions (zinc(II), iron(II), and nickel(II)). We further explore the transition from linear to nonlinear rheological behavior.

## 2. Experiments

Preliminary results with these systems have previously been published [7]. The polymer is a polystyrene-*b*-poly(*tert*-butyl acrylate) (PS-*b*-PtBA) block copolymer with average polymerization degrees of 80 (*M*_n_ ≈ 8300 g/mol) and 200 (*M*_n_ ≈ 25,600 g/mol) for the PS and PtBA blocks, respectively, and a polydispersity index of 1.11 for the whole copolymer. The end of the PtBA block is functionalized with a terpyridine ligand. The diblock copolymer forms micelles in ethanol, with a glassy PS core and PtBA corona. As determined by static light scattering, these micelles have a radius of gyration of approximately 15 nm and contain 28 chains on average. Metal ions can be added to this suspension of hairy colloids following a procedure described elsewhere [7]. Up to two terpyridine ligands form complexes with the divalent metal ions used in this article (Fe^2+^, Ni^2+^, and Zn^2+^), leading to supramolecular bonds either between chains from different micelles (forming crosslinks), or from the same micelle (forming loops) [7]. Three metal ions (Zn^2+^, Fe^2+^, and Ni^2+^) are added as chloride salts. The quantity of ions is half an equivalent with respect to the terpyridine [7].

Small-angle x-ray scattering (SAXS) experiments were performed on station DUBBLE at the European Synchrotron Radiation Facility, Grenoble, France. The X-ray wavelength was *λ* = 1.55 Å. SAXS patterns were collected with a two-dimensional multiwire gas-filled detector. The wavenumber scale (*q* = 4π × sinθ/*λ*, where 2θ is the scattering angle) was calibrated using a sample of wet collagen (rat tail tendon).

The rheological characterization was performed with a TA Instruments ARES (New Castle, DE, USA) using either a 25 mm/0.02 rad cone and plate geometry, a Malvern Kinexus (Malvern, Cambridge, UK) using a 25 mm/0.5° cone, or a TA Instruments AR-G2 (New Castle, DE, USA) using a 40 mm/2 or a 20 mm/1° cone. The measurements were carried out at room temperature in an ethanol-saturated atmosphere to minimize evaporation of the solvent. All measurements were carried out at 20 °C. Before measurement, an equilibration time of around 10 min was applied, which was found to be sufficient for all conditions. Dynamic frequency sweeps and nonlinear dynamic strain sweeps were also performed. 

## 3. Results

### 3.1. Structure of the Micellar Network

**Figure 1:**
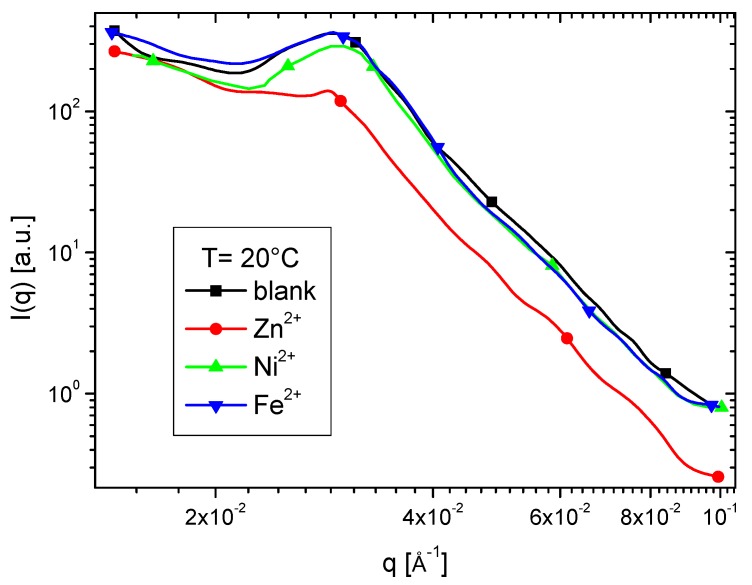
SAXS data collected on the investigated PS-b-PtBA micellar solutions in ethanol at a concentration of 12 w/v%.

To explain the rheological data, the results of the analytical characterization are essential. Figure 1 shows the SAXS data collected for the investigated samples at a concentration of 12 w/v%. For all ions, a clear peak around *q* = 0.03 Å^−1^ is observed, which corresponds to a long period of 27 nm and has been ascribed to intermicellar distance (i.e., the core-to-core distance *d_cc_*). Figure 1 also shows that no other order is present, which indicates that the micelles are organized in a liquid-like fashion. The value of *d_cc_* for the 15-w/v% sample is calculated. Considering the relatively low concentration and poorly ordered properties, we assume micelle spheres are homogeneously dispersed in solutions by simple cubic packing. Thus, *d_cc_* can be calculated as

(1) $$d_{cc}=d_{cc,\exp}\sqrt[{3}]{\frac{c_{\exp}}{c}}$$

where *d_cc_*_,exp_ and *c*_exp_ are experimentally obtained values (i.e., *d_cc_*_,exp_ = 27 nm for *c*_exp_ = 12 w/v%). For the sample with *c* = 15 w/v%, *d_cc_* is calculated as 25 nm. 

It is known from dynamic light scattering that the hydrodynamic radius *R_h_* of an isolated micelle is around 20 nm [7]. Static light scattering has been used to measure the radius of gyration of these micelles, determining a value of 15 nm [7]. The ratio between the radius of gyration and the hydrodynamic radius is, therefore, 0.75—a value close to the one expected for ideal spherical micelles (0.78). An ideal chain packing for the glassy PS cores leads to a core diameter of 9.1 nm. Considering packing errors and the slight swelling of the cores due to very small fractions of incorporated PtBA and ethanol, we assume that the “real” diameter is rather around 10 nm (core radius *R*_c_ = 5 nm). The number of chains per micelle on average is 28 (measured by light scattering) [7]. 

We assume that the micelles are equal in diameter and spherical. This is confirmed by TEM [15] and is logical considering the polymer concentration and block molecular weight (*M*_w_) ratio. The overlapping volume *V*_0_ of two equally sized spheres is given by [31] 

(2) $$V_{0}=\frac{1}{12}\pi (4R_{h}+d_{cc}){\left(2R_{h}-d_{cc}\right)}^{2}$$

Using the volume of the corona *V_c_* = 33,000 nm^3^, which is calculated as the difference of micelle and core volumes (*V*_micelle_ − *V*_core_), the overlapping fraction of one micelle with one neighbor *f*_1_ can be calculated as

(3) $$f_{1}=\frac{V_{0}}{V_{c}}$$

The relation of *f*_1_ and concentration (*c*) are determined by combining Equations (1)–(3).

**Scheme 2:**
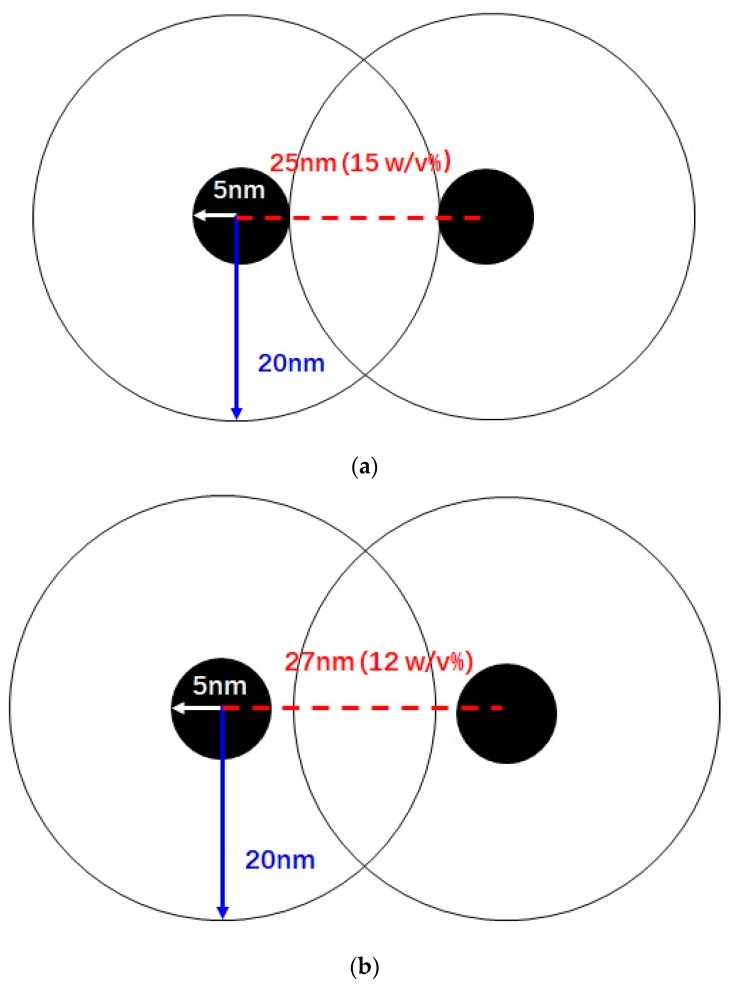

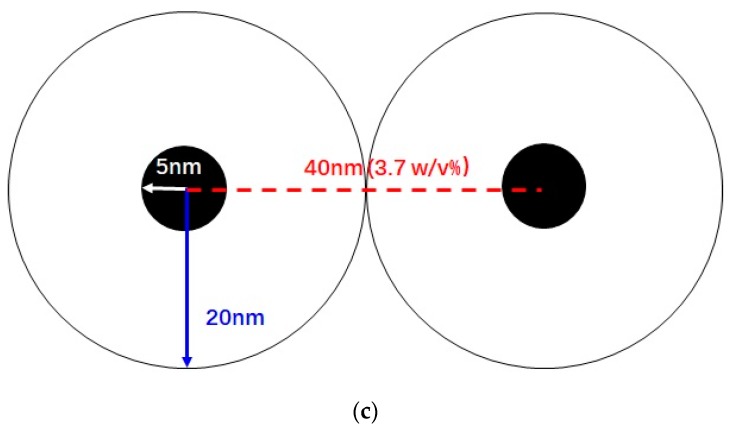
Cartoon of the size and distance of micelles (solid small circle representing core and unfilled large circle representing corona) at (**a**) concentration for total overlap of corona (15 w/v%); (**b**) concentration for partial overlap (12 w/v%); and (**c**) overlap concentration (3.7 w/v%).

Two coronae totally overlap when the micellar core of one micelle just touches the corona of the neighboring one (i.e., when *d_cc_* ≤ 25 nm (Scheme 2a)), which occurs around our concentration of 15 w/v%. For the 12-w/v% sample, coronae partially overlap (Scheme 2b). However, with dilution the overlap concentration at which the two coronae start to touch (Scheme 2c) reduces to as low as 3.7 w/v%, calculated from Equation (1). Moreover, the weak scattering signal indicates the system is very disordered (Figure 1). 

**Figure 2:**
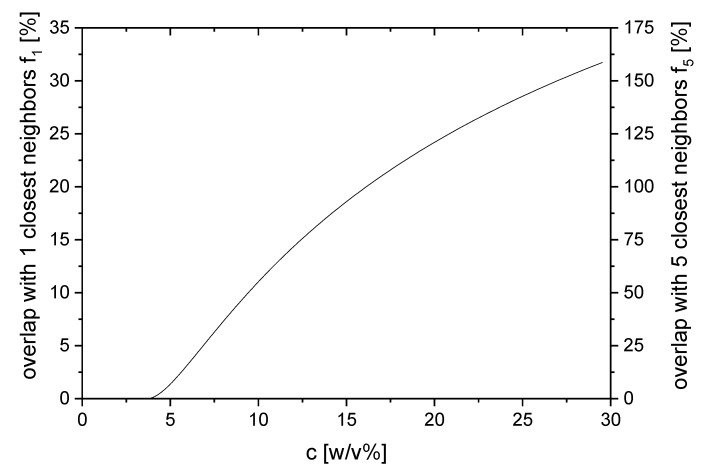
Overlapping fractions *f*_1_ and *f*_5_ as a function of concentration.

The overlapping fraction *f*_1_ is plotted as a function of concentration in Figure 2 (left *y*-axis). The value of *f*_1_ is null until 3.7 w/v%, then increases smoothly beyond that, suggesting that below a certain threshold no network can form because the micelles do not overlap and, hence, cannot form any intermicellar connection [32,33,34].

The disordered nature of the packing observed from the SAXS data (Figure 1) indicates that close packing is impossible and even cubic structures can hardly be reached. This puts the maximum number of closest neighbors below six (simple cubic packing). We reasonably assume that each micelle has about five closest neighbors, an assumption that has the best agreement with experimental data (see below).

Under the assumption of five closest neighbors, the combined overlapping fraction *f*_5_ (calculated from additive behavior of five neighboring micelles (*f*_5_ = 5 × *f*_1_)) is 100% at a 16-w/v% concentration and reaches 72% at 12 w/v% (Figure 2, right *y*-axis). Of course, the additive overlap assumption is too simple, as multiple overlaps in the same area can occur and complicate the picture, but it is a reasonable guideline and a fully quantitative analysis would require detailed knowledge of the packing, which cannot be gathered without 3D simulations of the structure. Based on the overlapping volume fraction, the volume fractions involving chains originating from one, two, and three different coronae (defined as *n*_1_, *n*_2_, and *n*_3_, respectively) can be calculated as

(4) $$\begin{array}{l} n_{1}=1-n_{2}-n_{3}\\ n_{2}=\left\{ \begin{array}{cc} f_{5} & \mathrm{when}\;c<16\;w/v\%\\ 1-n_{3} & \mathrm{when}\;c>16\;w/v\% \end{array}\right.\\ n_{3}=\left\{ \begin{array}{ll} 0 & \mathrm{when}\;c<16\;w/v\%\\ f_{5}-1 & \mathrm{when}\;c>16\;w/v\% \end{array}\right. \end{array}$$

**Figure 3:**
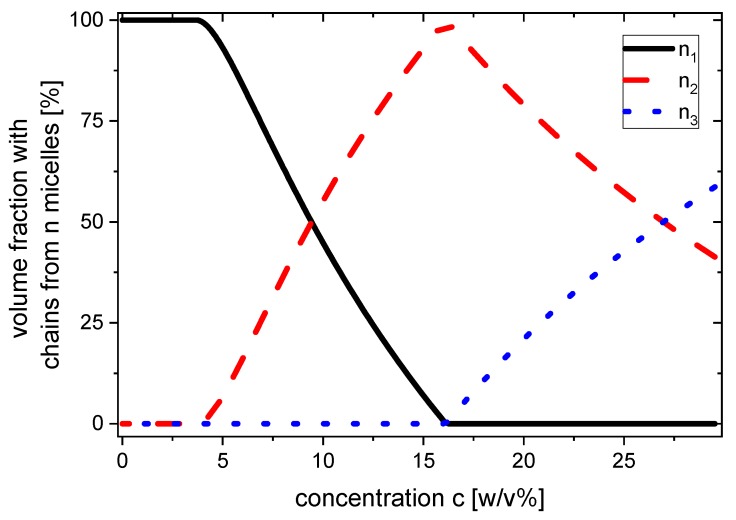
Volume fraction of non (*n*_1_), binary (*n*_2_), and triple (*n*_3_) coronae overlap as a function of concentration.

Their results are plotted in Figure 3. Up to about a 16-w/v% concentration, non-overlapping volume is present (*n*_1_), while above 16 w/v%, *n*_1_ becomes null but an overlap of three coronae (*n*_3_) appears. The value of *n*_2_ increases first with concentration but decreases beyond 16 w/v% due to the occurrence of a triple overlap.

Although a quantitative estimation has been made above, it is, regrettably, not possible to calculate the exact overlap, as we do not know how to account for the concentration difference between overlapping and non-overlapping regions of the coronae. There are two effects that cannot be disseminated without making a number of assumptions. First, it is clear that when two coronae overlap, the concentration is twice as high inside this overlapping area as outside. Because of this, there is a weak driving force, which will drive the chains out of the overlapping area. However, it is expected that the contribution will be small. Second, the exact geometry of the assembly is unknown and underlies statistical fluctuations. We know from the calculations that five closest neighbors are the most likely packing. However, it is not a disordered crystal but a random structure with average core-to-core distances underlying statistical fluctuations. As a result, based on the assumptions being put into Figure 3, we can estimate that a probable 100% overlap (i.e., the disappearance of *n*_1_) is reached close to a concentration of 20 w/v%. This number is somewhat higher than Figure 3 suggests, as we have to assume that the overlap does not occur with perfect homogeneity.

Please note that in this simple approach it is assumed that no overlap of three coronae can occur so long as non-overlapping coronae are still present. In reality, the situation evolves more progressively, which will smooth the curves somewhat. The effect of this simplification, however, should be small. For example, we have to assume that, in reality, the disappearance of *n*_1_ and the appearance of *n*_3_ does not occur at 16 w/v%, but rather at a certain interval where both areas have no overlap (*n*_1_) and where with three overlapping coronae (*n*_3_) coexist due to geometric reasons. This is also the reason why we have to assume that, as mentioned in the previous paragraph, 100% overlap is reached at slightly higher concentrations than Figure 3 indicates.

One might argue that, according to the model of Daoud–Cotton [35], the density of chains close to the surface of the micellar core is higher than that in the outer areas of the micellar shell. The fact that the chains are only at 28% of their stretched length, which corresponds to the approximate value of free chains (not bound in a micelle) in solution is a clear indicator that the connection of the PtBA arms to the micellar core does not play a major role in coil size. Hence, we can also conclude that no distinct concentration gradient is present in micelle coronae. This sets the micellar gels in this article apart from other soft colloids (e.g., multi-arm stars) [35,36].

If we reasonably assume that the overlapping fraction based on five closest neighbors determines the amount of potential supramolecular crosslinkers in the system, it becomes possible to calculate the fraction of crosslinks that should exist between chains from different micelles (intermicellar links, *f_link_*) with the help of Equation (5).

Here, we assume that two end-functionalized chains can 100% link considering the high bonding strength of terpyridine and Ni^2+^/Fe^2+^. Besides, the chains should not repel each other. These assumptions should work safely at relatively low concentrations, as we have here. Hence, there are two possibilities for the bonding partners, chains from other micelles (intermicellar bond) and chains from the same micelle (intramicellar bond). Under overlap, the intermicellar bonding probability is 0% when no overlap is present (*n*_1_), 50% (1/2) when two micellar coronae overlap (*n*_2_), and 66.67% (2/3) when three micellar coronae overlap (*n*_3_). This simple probabilistic analysis neglects conformational free-energy differences between the intra- and intermicellar bonds. The outcome is the fraction of intermicellar links *f_link_*:

(5) $$f_{link}=\frac{\frac{1}{2}n_{2}+\frac{2}{3}n_{3}}{n_{1}+n_{2}+n_{3}}$$

**Figure 4:**
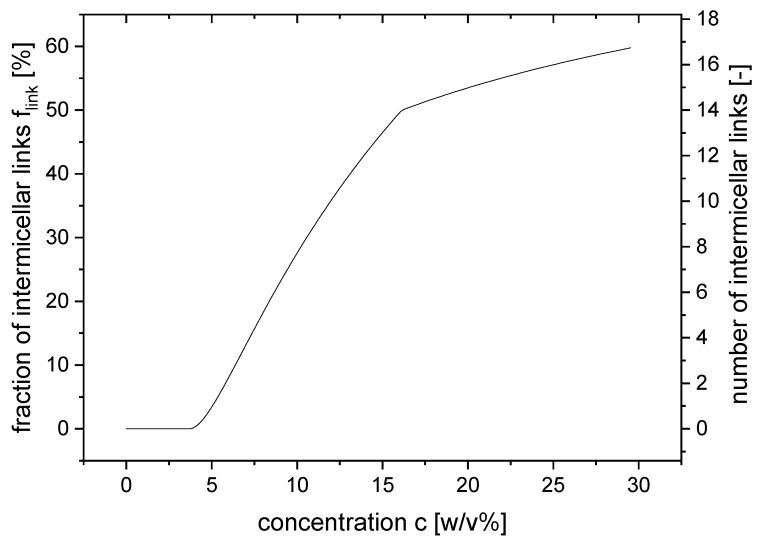
Average fraction and number of intermicellar links as a function of concentration.

Figure 4 shows the fraction of intermicellar links *f_link_* as a function of concentration. It becomes obvious that for smaller concentrations than 3.7 w/v%, only a negligible fraction of intermicellar links can exist (barely enough for a linear “train” of micelles), while for concentrations around 16 w/v%, a 50–50 share of inter- and intramicellar crosslinks is found. The reason for the sudden change in slope around 16 w/v% is the disappearance of non-overlapping volume. The fraction of intermicellar links for a given micelle is directly proportional to the total number of its intermicellar links (simply multiplied by the total number of ligand end-groups, 28 × *f_link_*). This is also plotted in Figure 4.

Qualitative tube inversion tests (i.e., the sample sticks to the bottom of the tube even when the tube is turned upside down), have shown that for concentrations around 9 w/v%, a weak but stable gel is formed when using Fe^2+^ or Ni^2+^ ions. When comparing this value to Figure 4, we see that a concentration of 9 w/v% roughly corresponds to six intermicellar links per micelle, which is numerically marginal to form a network. This is a strong indicator that the assumptions and calculations given above provide a reasonable prediction. 

### 3.2. Influence of the Type of the Ions and Polymer Concentration

**Figure 5:**
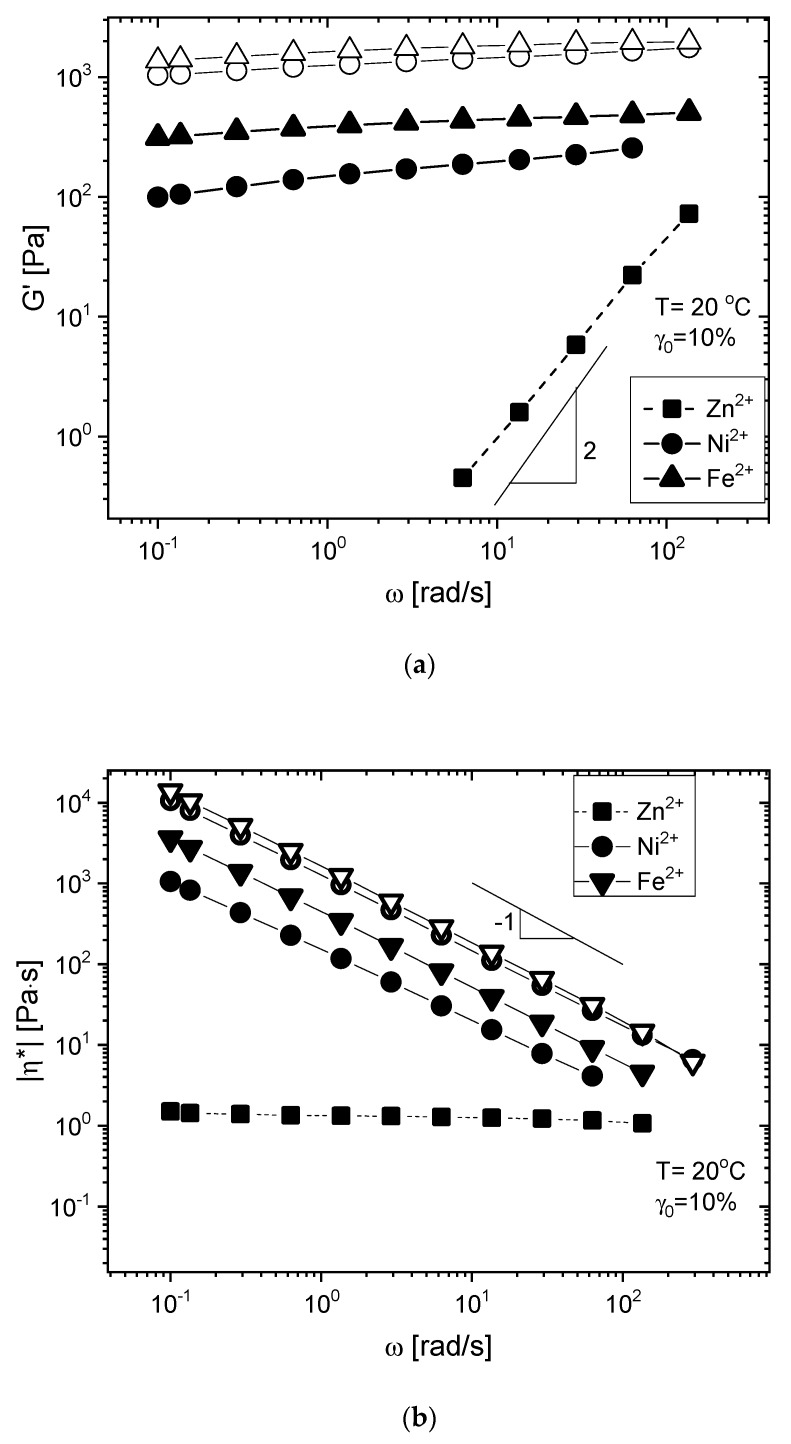
Frequency-dependent rheological data for (**a**) *G′*(*ω*) and (**b**) |η*(*ω*)| at *T* = 20 °C. Filled symbols: 12 w/v% concentration, from ref [7]. Open symbols: 15 w/v% concentration.

The frequency-dependent storage modulus and dynamic viscosity of colloidal suspensions are given in Figure 5 and Figure S1. Very diverse behaviors are observed depending on ion or concentration. The blank sample has very low viscosity (~0.03 Pa·s, roughly 30 times the viscosity of water) and no measurable elasticity. The 12-w/v% sample containing Zn^2+^-ions has a 40 times higher viscosity than the blank sample, showing slightly shear thinning and very weak but measurable elasticity. This sample thus shows only a minor departure from Newtonian behavior. In sharp contrast, the samples containing Ni^2+^ and with Fe^2+^ ions are characterized by much higher *G′*, and are dominated by their elasticity, since their storage moduli are significantly larger than their loss moduli (see the supporting information for *G″*(*ω*)). Furthermore, *G′*(*ω*) and *G″*(*ω*) are approximately constant over a wide range of frequencies. Such behavior is typical of a rubbery network. The complexes thus create (reversible) crosslinks between the micelles. As the elastic moduli are very low, these materials are best described as ultrasoft elastic gels. 

When the concentration is increased to 15 w/v%, the behavior of the blank sample stays roughly unaffected, while a significantly higher modulus is found for samples with ions. The trend of the data remains very similar to the 12-w/v% samples, except that *G′* and |η*(*ω*)| are higher by a factor of approximately 10. Although for Ni^2+^ and Fe^2+^ samples, the power law slope is close to 1 for |η*(*ω*)| and close to 0 for *G′*(*ω*), reflecting almost perfect elastic behavior, it is still slightly below that limiting level. Hence, none of the samples can be considered as a truly ideal rubber state.

**Figure 6:**
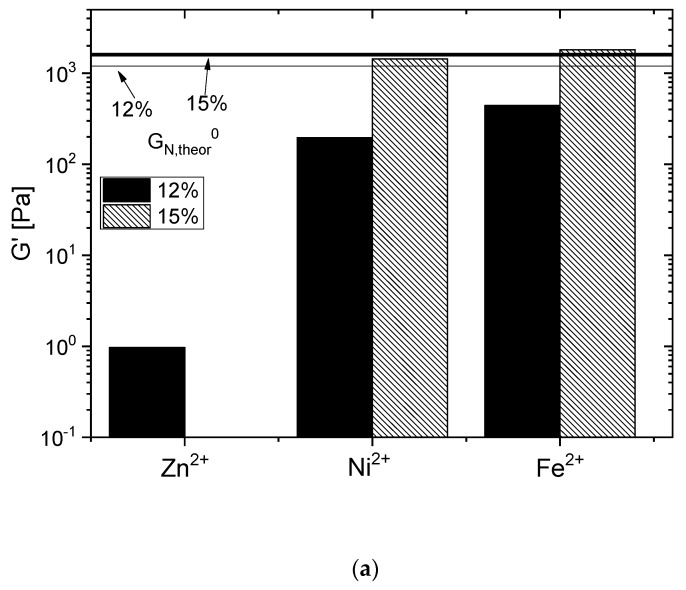

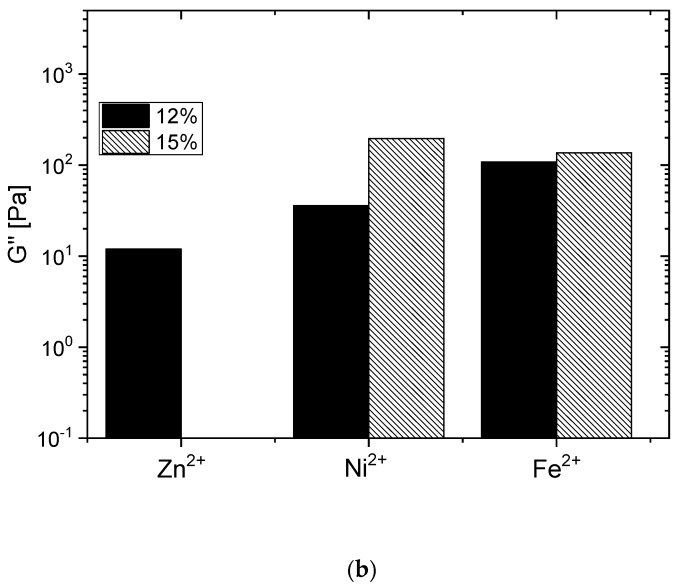
(**a**) Storage and (**b**) loss moduli, *G*’ and *G*”, at ω = 10 rad/s and *T* = 20 °C. The thick and thin horizontal lines are theoretical plateau modulus *G*_N,theor_^0^ for the 12- and 15-w/v% samples, respectively.

The ion nature has a profound influence on the strength of the gel, as can be observed by a comparison of the storage and loss modulus at a given frequency *ω* (10 rad/s) and concentration (Figure 6). Although comparison under the same Deborah number (*ω* × *τ*_d_) is also widely used for samples, the terminal relaxation time *τ*_d_ is not experimentally reached here, and we mainly focus on the plateau moduli that reflects the network density. Therefore, the comparison at a given *ω* is fair, considering the *G′* plateau is broad enough to be approximately frequency independent. As reported above, Zn^2+^, at a 12-w/v% concentration only leads to a 40-fold viscosity increase in respect to the blank. At the same concentration, and unlike the Zn^2+^ sample, the Ni^2+^ sample withstands the tube inversion test. Rheologically, this is understood as a gelation. By comparing with Zn^2+^, Ni^2+^ leads to an increase of *G′* by a factor of 600 and makes *G′* larger than *G″* (see Figure 6). Fe^2+^ leads to a further increase of *G′*. Thus, Ni^2+^ and Fe^2+^ samples both have a rubber-like behavior, but are much softer than a conventional elastomer due to the static dilution by solvent, leading to a low crosslinking density. Hence, this behavior is best described by the term “soft rubber”. The different rheological behaviors among three ions are due to the strength of the complexes. Since Zn^2+^ forms relatively weak complexes with terpyridines, Fe^2+^ and Ni^2+^ form much stronger complexes that are difficult to disassociate [37].

Going from a 12- to 15-w/v% concentration essentially has negligible effect on the viscosity of the blank. The behavior of the 15-w/v% samples loaded with ions, however, is distinctly different from their 12% counterparts in terms of the moduli and different nonlinear behavior discussed later. For the Zn^2+^ containing the 12-w/v% sample, *G″*(*ω*) is higher than *G′*(*ω*), while *G″*(*ω*) is lower than *G′*(*ω*) for the Ni^2+^/Fe^2+^ containing 12-w/v% samples. For the 15-w/v% Fe^2+^/Ni^2+^ samples, *G′*(*ω* = 10 rad/s) is almost independent of the ion used, with all values around 1800 Pa. However, differences are still clear for *G″*(*ω* = 10 rad/s). These results indicate that a threshold is reached for the storage modulus at a 15-w/v% concentration. Unlike the 12-w/v% samples, the 15-w/v% samples behave like a solid as soon as ions are added. The stronger Ni^2+^- and Fe^2+^ supramolecular bonds lead to a more pronounced elastic behavior characterized by a lower phase angle δ (tan δ = *G″*/*G′*). The most likely mechanism is that the number of unlinked chain ends is lower because the equilibrium constant for complex formation is higher. Based on investigations on the lifetime and association constant of ion–terpyridine complexes by Dobrawa and Würthner [38], we can conclude that, at equilibrium, the vast majority of the terpyridine end groups are actually complexed by the ions in the Ni^2+^ and Fe^2+^ samples.

### 3.3. Entanglement Molecular Weight and Plateau Modulus

The next interesting question is how many of the bonds are intermicellar or intramicellar? This can be estimated by comparing the experimental plateau modulus *G*_0,exp_ of these samples to predictions obtained from the structural model outlined above.

Linear viscoelasticity rubber theory is only correct *stricto sensu* if we assume that we have a permanent homogeneous network without loops or dangling ends. Moreover, entanglements between network strands can complicate the picture. The first condition is not fully met by our supramolecular systems because of the transient nature of the bonds and existence of intramicellar links beside intermicellar ones. Hence, the elastic moduli must be lower than the theoretical values. Stable intermicellar bridges can be assumed for the Ni^2+^ and the Fe^2+^ samples but not for the 12-w/v% Zn^2+^ and blank sample. The second condition (no entanglements) is true if the dilution is so high that the chains are unentangled. An estimation of entanglement molecular weight (*M_e_*) under dilution can be obtained from the following equation [39]:

*M_e_* = *M*_e,bulk_/*ϕ*^α^ (6)

where *ϕ* is the polymer volume fraction in solution, *α* = 1.3 is the dilution exponent, and *M*_e,bulk_ is the entanglement molecular weight in a bulk state. According to Fetters et al. [40] and Tong et al. [41], *M*_e,bulk_ of alkyl acrylate polymers is in the range between 7700–15,200 g/mol. However, the value of poly(*tert*-butylacrylate) is not given. Based on the available literature data [40,41], it is estimated (based on the bulkiness of the side chains and the established *M_e_*-values) that *M*_e,bulk_ (PtBA) is in the range of ~12,000 g/mol. Because the polystyrene chains are in the glassy rigid core, they do not play a role here. Using 0.79 g/cm^3^ as density for ethanol and 1.07 g/cm^3^ as density for the PS-PtBA copolymers, we find that a 12-w/v% fraction is equivalent to the volume fraction *ϕ* = 0.156 and that, similarly, a 15-w/v% fraction corresponds to *ϕ* = 0.193. As a result, the diluted *M_e_*, as calculated from Equation (6), is 134,000 and 102,000 g/mol for 12 w/v% and 15 w/v%, respectively. Both values are much larger than a closed elastic strand (i.e., two supramolecularly connected arms (2 × *M*_arm_ ≈ 51,200 g/mol). Hence, physical entanglements among corona chains do not play a significant role for our samples and we will only discuss the quasi-permanent crosslinks—intermicellarly crosslinked PtBA-chains—in the following. 

We can now estimate the theoretical elastic modulus *G*_0,theor_ of our supramolecular systems, viewed as permanent networks, by using the equivalent crosslinking density predicted from Figure 4:

(7) $$G_{0,\mathrm{theor}}=nRT$$

where *n* is the elastic strand density comprising intermicellarly connected PtBA arms. The values of *n* are obtained by dividing the number of intermicellar bonds of a given micelle by the volume pervaded by arms (the volume of corona; i.e., 28 × *f_link_*/*V_c_*). The values of *G*_0,theor_ are thus calculated based on *n*. Both *n* and *G*_0,theor_ are shown in Table 1.

A comparison between predicted and experimental values for the moduli at *ω* = 10 rad/s (Table 1 and Figure 6) shows that for the 12-w/v% samples the experimental modulus is lower by a factor of about 2.7 versus the theoretical one for the Fe^2+^ sample, and even lower for the other samples. For the 15-w/v% samples, values between theoretical and experimental results are almost quantitatively consistent. At higher frequencies, undoubtedly higher moduli would be present, which, however, cannot be measured reliably, because of inertia effects.

Although the simplified geometric model only provides an approximation of the maximal crosslinking density, it is clear that the behavior of the 12-w/v% and 15-w/v% samples is different. The ratio of 2.7 for the 12-w/v% samples indicates that the majority of the supramolecular crosslinkers are either open (i.e., free chain ends) or do not contribute to the crosslinking network (i.e., closed loops, presumably corresponding to intramicellar supramolecular bonds). Isolated groups of micelles crosslinked with each other can also be responsible for the lower effective experimental crosslinking density. However, it is expected that for the samples discussed here, this effect is negligible. 

*G*_N_ is proportional to the elastic strand density comprising intermicellarly connected arms. Hence, only about 37.5% (1/2.7) of the predicted intermicellar bonds in the 12-w/v% sample actually contribute to the network strength for the Fe^2+^ sample. For the other samples, this ratio is even lower. The phase angle δ ≈ 11° for the Ni^2+^ and the Fe^2+^ samples implies a relatively low amount of free chain ends. For the ion-treated 15-w/v% samples, almost all of the predicted intermicellar bonds contribute to the network. Actually, this result applies only to crosslinkers stable at the time scale of the experiments (measured for aqueous solution with a time of stability for a Fe^2+^ and Ni^2+^ ions in the order of 30 min, according to the literature data [38]). For shorter times (corresponding to higher frequencies), a higher modulus would presumably be found and, thus, a higher proportion of closed crosslinkers would be present.

Such a significant difference in elastic behavior for a minor concentration difference (from 12 to 15 w/v%) is rather unexpected and is indicative of a major modification of the supramolecular structure. For the 15-w/v% sample with sufficiently overlapped coronae, the consistency between predicted and experimental *G*_N_^0^ confirms our approximation that half (exactly 46% according to Figure 4) of terpyridine end groups link intermicellarly, while another half link intramicellarly. This also implies that the fraction of the open terpyridine end groups is negligibly small. For the 12% sample, the same argument does not yield such a nice agreement. Assuming a statistical distribution of the terpyridine end groups, 36% of the arms are predicted to intermicellarly link (see Figure 4), which is significantly higher than (although qualitatively consistent with) the value of 13.5% (=36% × 37.5%) suggested by the experiment.

This deviation of 12-w/v% samples can be explained by two factors. Firstly, the fact that some areas of the corona volume overlap, while others do not, means that the distribution of chains is not homogeneous and, hence, some micelles may not participate in the percolating elastic network. This effect can further be compounded by the following argument. The connected chains will be somewhat stretched out of their equilibrium configuration after making the bond, because the resulting supramolecular assembly will suddenly be tethered on two surfaces instead of one. Hence, they will pull the micelles together while trying to get back to equilibrium. This is likely to exacerbate local concentration heterogeneities, an effect that will be much more pronounced for the 12-w/v% sample as the overlapping fraction of neighboring micelles is only 72% (i.e., 28% of the micellar corona is not shared) when not taking the inhomogeneities into account, which will increase this fraction. The consequence of this is that the non-overlapped parts of the corona are not pulled by neighboring micelles, while overlapping coronae tend to influence each other and get closer to each other due to thermodynamic reasons. As the 15-w/v% sample practically does not contain any non-overlapping coronae fractions, this effect is much less pronounced, although it can be quite severe at lower concentrations. Secondly, the average distance measured by SAXS is a number average, but the average intersecting volume does not scale linearly with distance. If the distance between the spheres is polydisperse, the average overlapping volume will deviate locally from the value calculated from the average distance. For the current conditions, there is probably a somewhat smaller overlap than the one calculated from the average size.

### 3.4. Strain Dependence

**Figure 7:**
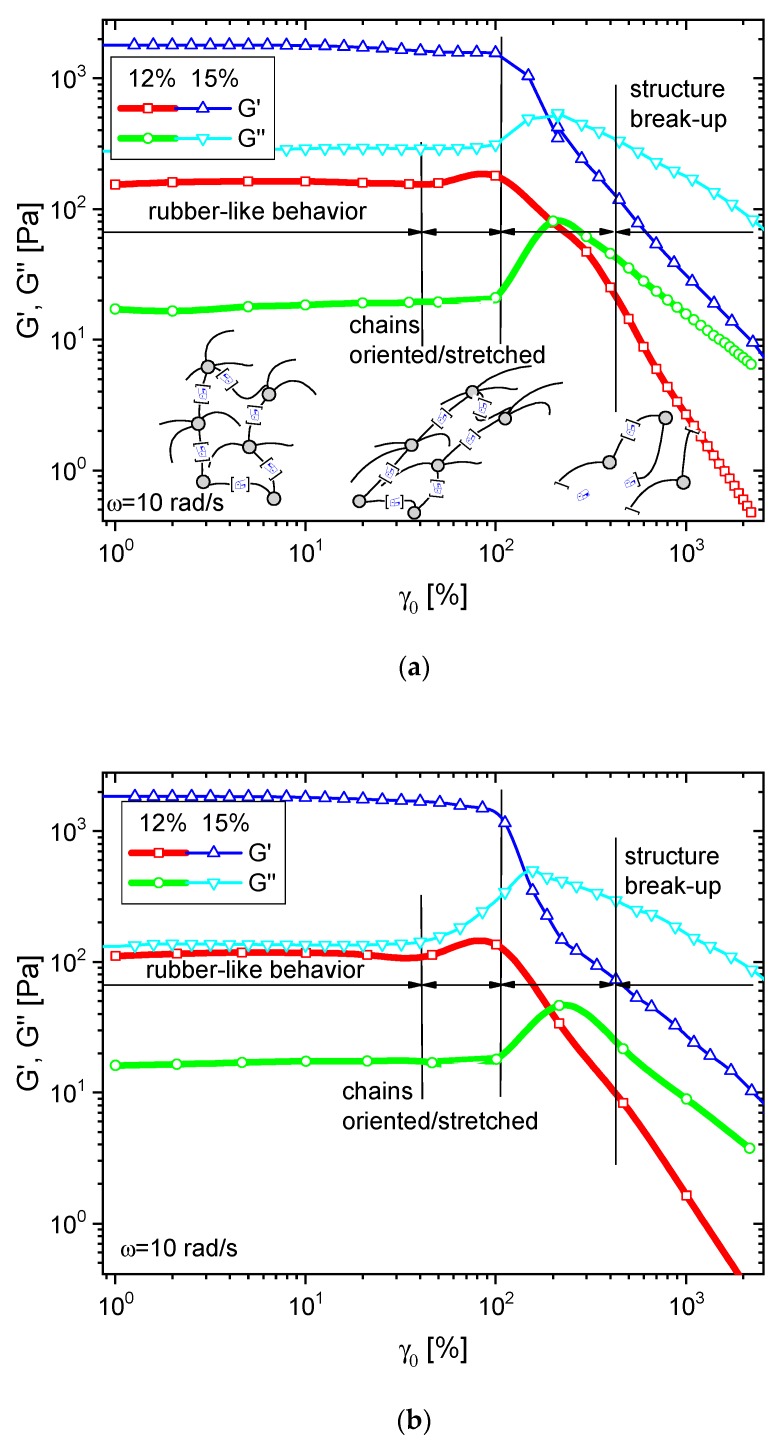

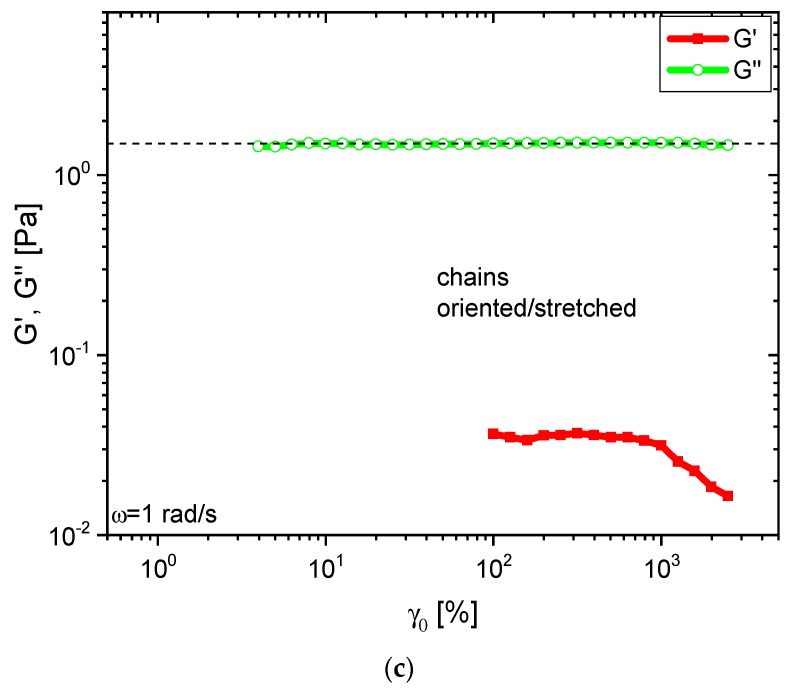
Strain sweep of (**a**) 12-w/v% Ni^2+^ (from ref [7]) and 15-w/v% Ni^2+^ samples; (**b**) 12-w/v% Fe^2+^ and 15-w/v% Fe^2+^ samples; and (**c**) the 12-w/v% Zn^2+^ sample.

The strain dependence of Fe^2+^, Ni^2+^, and Zn^2+^ samples at a given angular frequency *ω* is presented in Figure 7a–c. The behavior is qualitatively similar between Fe^2+^ and Ni^2+^ samples at the same polymer concentration. Below 100% deformation, *G′*(*ω, γ*_0_), *G″*(*ω, γ*_0_), and hence tanδ remain constant, which means that the system is still in the linear viscoelastic regime. Moreover, the loss factor is distinctly below 1, indicating an elastic-dominated behavior. At a strain around 100%, an upturn in *G′*(*ω, γ*_0_) is evident for the 12-w/v% sample, however, not for the 15-w/v% sample. At a higher strain *γ*_0_ around 300%, an even stronger peak in *G″*(*ω, γ*_0_) is found, which is very close to the *G′* and *G″* crossover point *ω_c_*(*γ*_0_). This crossover point reflects the transition from elastic to plastic behavior [42,43,44,45]. We note that the dynamic moduli reflect average responses, and that the instantaneous responses could show dramatic differences and yet still have the same average value. At higher strains, *G′*(*γ*_0_) and *G″*(*γ*_0_) for the 12-w/v% sample decrease with slopes of 2 and 1, respectively. This corresponds to a sharp increase of tanδ (*γ*_0_). 

This feature combining an upturn in both *G′* and *G″* during the transition to nonlinear behavior has been rarely reported. It is described as strain sweep type IV by Sim et al. [46]. Most of the reports of a type IV strain sweep stem from characterization of semidilute supramolecular/associative solutions [47], including hydrophobic ethoxylated urethane (HEUR), hydrophobically modified alkali-swellable associative polymer (HASE), and similar systems [47,48]. Annable et al. [49] and Tanaka and Edwards [50,51,52] analyzed these findings from a theoretical point of view. In general, the behavior can be explained with the help of a transient network. The network is stretched until a certain stress is exerted on the chains. When that critical stress is reached, the supramolecular bond breaks and the behavior changes from elastic to plastic-dominated. For the 12-w/v% samples, the critical stress is around 70 Pa for the Ni^2+^ sample and 180 Pa for the Fe^2+^ sample. At higher strains, the stress remains approximately constant at this level (Figure S2), as can be inferred from the −1 slope of the *G″*(*γ*_0_) in the nonlinear region (see Figure 7a). Based on literature [46,47,48,49], the stretching (peak in *G′*(*γ*_0_)) only occurs in a certain concentration regime. Therefore, a certain degree of dilution is needed to find the chain stretch. This stretching appears to only occur when the chains can be stretched to a certain degree before breaking the first bonds, which depends on the average distance between the micellar cores (as discussed later). It seems that this regime is reached for the 12-w/v% samples but not for the 15-w/v% samples.

For the 12-w/v% Zn^2+^ sample (Figure 7c), an almost perfectly linear behavior is found up to deformations of *γ*_0_ ≈ 1000%. Above this threshold, a clear decrease in *G′*(*ω*) and a weak decrease in *G″*(*ω*) is found. This indicates that the Zn^2+^ sample has a very weak structure that can only be broken at a very high strain, suggesting that this structure exists in clusters of only very few micelles. This does not mean that the material consists of small stable clusters in a liquid. Instead, weak dynamic interactions exist among different micelles, which connect several micelles at a certain point in time. The cluster, however, is dynamic (i.e., the micelles involved in it change). This concept is similar to sticky reptation [2].

The structure of the 12-w/v% samples can be understood from the schemes inserted in Figure 7a. At a low strain *γ*_0_ the micelles are connected by the supramolecular bonds (indicated by ●), forming a network. At strains around 50%, the intermicellar links become oriented and stretched, which leads to an increase in the storage modulus *G′*(*ω*, *γ*_0_). At a somewhat higher strain *γ*_0_, *G″*(*ω*, *γ*_0_) also increases distinctly, reflecting higher dissipation due to a massive breakup of the intermicellar links, which can also be concluded from the decrease of *G′*(*ω*, *γ*_0_). At even higher deformations, the number of intermicellar links is reduced so much that the network breaks down, which leads to a behavior typical of polymer solutions and melts.

The transition to nonlinear behavior of the 15-w/v% samples is significantly different from that of the 12% samples, despite the minor difference in concentration. For the former, the distance between the cores is shorter. This leads to a larger overlap of the coronae and, thus, to a higher likeliness of intermicellar bonds. This in turn increases the likeliness of twists and contact of the intermicellar “hairs”, which self-evidently leads to lower stretchability before breaking. A close look at Figure 7a,b reveals that the onset of non-linearity is around 40% for both samples. However, contrary to the 12-w/v% samples, there is no *G′*(*γ*_0_) upturn for the 15-w/v% samples (this corresponds to “type III” behavior in the nomenclature of Sim et al. [46]). This difference can be interpreted by assuming that the 15-w/v% samples already show the first signs of network breakdown before they get a chance to be stretched. At very high strains (*γ*_0_ around 300%), a constant slope is reached for the loss factor (*G″*/*G′*) of both samples, which is lower for the 15-w/v% sample than for the 12-w/v% sample. Hence, the former sample structure degrades faster. This can also be inferred from the different negative slopes observed for *G′*(*γ*_0_) and is logical considering the much higher micellar overlap of the 15-w/v% sample, leading to much higher network “healing” probability under flow.

From micellar inter-distance and the chain length of the acrylate blocks, it is possible to estimate the maximum stretching before breakage. The calculations were made assuming that the cores are rigid and that the chain is tethered perpendicular to the shear plane initially. The equilibrium length of connecting strands is the distance between surfaces of two neighboring micelles (i.e., 17 nm for the 12-w/v% sample and 15 nm for the 15-w/v% sample). If the strand is stretched to a maximum extent, the strand length is the contour length of 2 × 200 monomers. By approximating the length of a covalent bond as 0.154 nm, the maximum stretch leads to 2 × 200 × 0.154 nm = 61.6 nm. Hence, for a 12-w/v% concentration, the theoretical stretching limit is at 61.6 nm/17 nm = 360%, while 61.6 nm/15 nm = 410% is possible for a 15-w/v% concentration. Comparison of this threshold with the data in Figure 7 suggests that there is a link with the microscopic nonlinear behavior dominated by dissipation (constant positive slope tanδ region).

As for the contribution of cores, although the type of large amplitude oscillatory shear (LAOS) behavior is affected by colloidal particles [53], PS core has a much smaller size than the coronae, and the concentrations of PS cores are rather low. Therefore, the PS core is similar in its function to a crosslinker and has only a limited effect on LAOS types at the studied concentrations. A study of the core’s effect on LAOS could be realized by either increasing the concentration of polymers or tuning the relative length between core and coronae chains. This is beyond the present topic and prohibited by limited samples, but deserves systematic research in the future.

At last, we note that rheology combined with simultaneous scattering could be an effective tool to clarify the structural evolution under large-amplitude strain. This technique has been widely used to characterize the phase behavior of block copolymers during shear [54]. Specifically, for less ordered systems as ours (e.g., solutions, gels, and suspensions), a lot of literature focuses on in situ research of the alignment of block polymers under transient and steady shear [55,56,57,58,59,60,61,62,63,64,65,66]. However, the research on the associative colloidal polymer assemblies is still missing as far as we know. We performed time-resolved SAXS measurements to characterize the present system, but the scattering was too weak to get valuable data, probably because the electron contrast between PS cores and ethanolic solutions of PtBA was rather low. Fortunately, the change of network under shear can be theoretically interpreted based on the classical model of Tanaka and Edwards [50,51,52], which addresses the orientation and stretching of strands in a network—even without further experimental evidence from scattering, a discussion based on such a model can be used in analyzing the nonlinear rheological behavior of micellar gels.

## 4. Summary and Conclusions

Linear and nonlinear rheological characterization combined with simple geometric considerations about micellar overlap fractions provides a very good insight into the structure and dynamic behavior of the studied micellar assemblies in the absence or presence of metallo–supramolecular interactions, depending on ion type and polymer concentration. 

In the linear regime, addition of Zn^2+^ only leads to viscosity increase for the 12-w/v% sample, but does not affect the Newtonian behavior. On the other hand, Ni^2+^ and Fe^2+^ induce a major shift towards an elastic dynamic network behavior because of semi-permanent intermicellar supramolecular interactions. Based on a comparison between theoretical and experimental elastic moduli combined with known concentrations of ligand-terminated corona chains, we conclude that only a 13.5% fraction of potential crosslinks are effective for network elasticity at a 12-w/v% polymer concentration, while 46% are effective at 15 w/v%. In the latter case, the experimental result is consistent with the theoretical prediction calculated by the intermicellar bond formation probability with the help of a simple geometric model using micellar overlap concepts. At a 12-w/v% polymer concentration, however, elastic strand density is much less than anticipated. Estimations of entanglement molecular weights of the solutions indicate that entanglements play no significant additional role in explaining the elasticity of the systems.

In the nonlinear rheological aspect, the Ni^2+^ and Fe^2+^ elastic networks show stretching at intermediate deformation (around 100%) and dynamic breaking of the supramolecular bonds at high deformation. The 15-w/v% samples start breaking down at a lower strain than the 12-w/v% ones, but demonstrate better reforming ability at large deformations because of higher micellar overlap.

Based on all results, the equilibrium structure of the gels can be pictured as soft interpenetrating spheres that are connected intermicellarly by less than 50% of the chains. The remaining chains either do not have a supramolecular bond or are linked intramicellarly and, thus, do not contribute to the network. The ratio between these two types of functional groups (inter- vs. intramicellarly bonded) depends on the polymer concentration. The ion used additionally determines how many of the potentially available groups are efficiently bonded. The low strength and lifetime of the Zn^2+^ ions [37] makes only a weak shear thinning fluid at a 12-w/v% concentration. The Ni^2+^- and Fe^2+^-based supramolecular bonds possess a much higher stability [37] and resulting micellar assemblies, and hence behave as very soft elastomers that could be self-healing [7]. One might argue that shear banding might be observed. However, as this would involve the collective and simultaneous break of all supramolecular bonds at the periphery, it is not a very possible process and even if it did occur the self-healing capability of the supramolecular bonds would quickly close it.

Samples at a 12-w/v% concentration show a small peak in storage modulus *G′* around 80–100% deformation. This can be explained by stretching of the supramolecular network, in agreement with some literature. The higher concentration 15-w/v% samples do not show this peak, which is explained by the fact that the network starts to break before it can be stretched, as the intensive twist and contact of intermicellar links make them less stretchable.

## Figures and Tables

**Table 1:** Plateau moduli calculated at *T* = 20 °C.

	12 w/v%	15 w/v%
*ϕ* [-]	0.156	0.193
*n* [mol/m^3^]	0.50	0.65
G_0,theor_ (Equation (7)) [Pa] ^a^	1200	1600
G_0,exp._(experimental, Fe^2+^-containing samples at 10 rad/s) [Pa]	450	1900

^a^ Assuming permanent bonds with infinite lifetime.

## Supplementary Materials

The following are available online at https://www.mdpi.com/2073-4360/11/10/1532/s1.
