# Supplementary material for: Linear and Nonlinear Dynamic Behavior of Polymer Micellar Assemblies Connected by Metallo-Supramolecular Interactions
Source: Polymers (Basel). 2019 Sep 20;11(10):1532. doi: 10.3390/polym11101532 (PMC6835675; doi:10.3390/polym11101532)
Supplement: Supplementary file 1 [file polymers-11-01532-s001.pdf]

Linear and nonlinear dynamic behavior of polymer micellar assemblies connected by metallo-supramolecular interactions

Zhi-Chao Yan,[a] Florian J. Stadler[a,c*], Pierre Guillet[c,d], Clément Mugemana[c,e], Charles-André Fustin[c], Jean-François Gohy[c], Christian Bailly[b]

[a] Shenzhen Key Laboratory of Polymer Science and Technology, Guangdong Research Center for Interfacial Engineering of Functional Materials, College of Materials Science and Engineering, Shenzhen University, Shenzhen 518060, China

[b] Université catholique de Louvain, Institut de la Matière Condensée et des Nanosciences, Bio and Soft Matter Division, Place Croix du Sud 1, B-1348 Louvain-la-Neuve, Belgium

[c] Université catholique de Louvain, Institut de la Matière Condensée et des Nanosciences, Bio and Soft Matter Division, Place Pasteur 1, B-1348 Louvain-la-Neuve, Belgium

[d] Université d'Avignon et des Pays du Vaucluse, Département de Chimie, 33 rue Louis Pasteur, 84000 Avignon, France

[e] Luxembourg Institute of Science and Technology (LIST), Esch-sur-Alzette, Luxembourg

*to whom correspondence should be addressed: fjstadler@szu.edu.cn

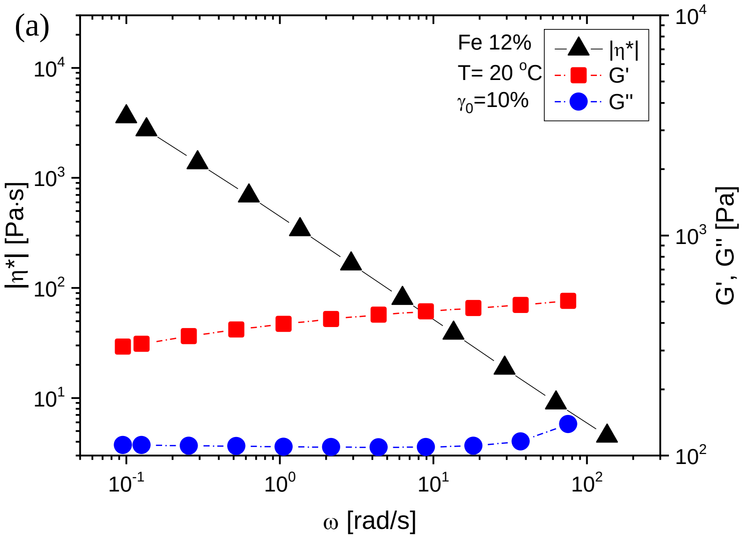

(b)

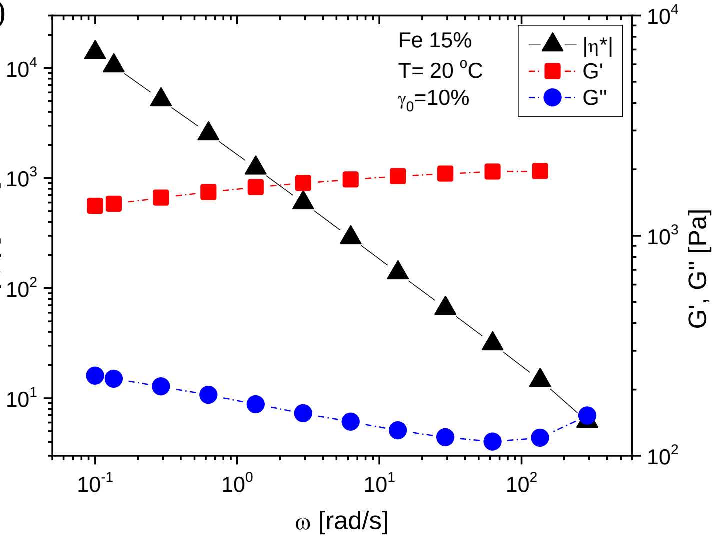
Fe 15%
T= 20 °C
γ0=10%
|η*|
G'
G''
|η*| [Pa·s]
G', G'' [Pa]
ω [rad/s]

(c)

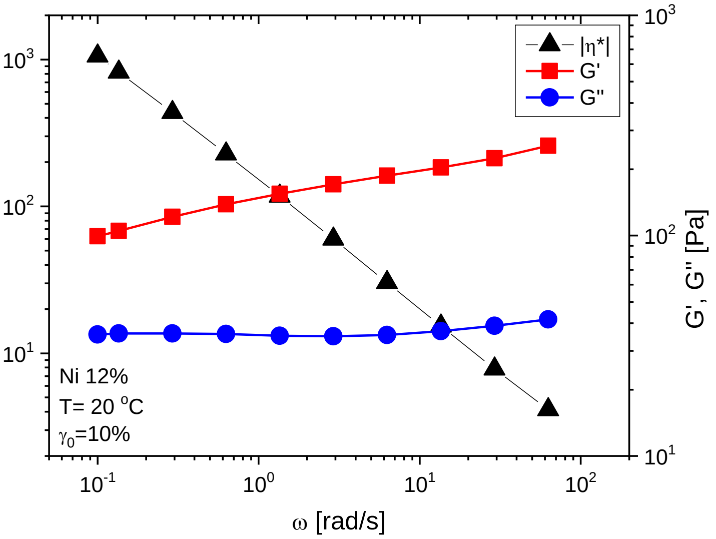
|η*|
G'
G''
Ni 12%
T= 20 °C
γ0=10%
|η*| [Pa·s]
G', G'' [Pa]
ω [rad/s]

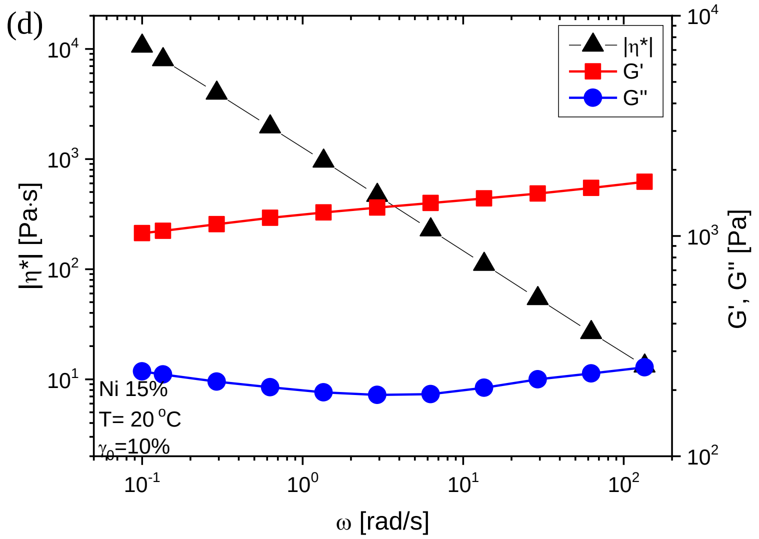

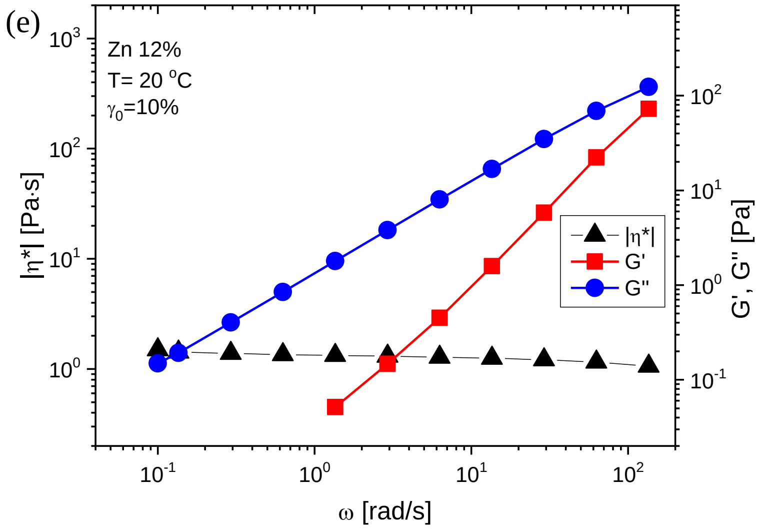

Figure S1. The storage (squre) and loss (circle) moduli and complex viscosity (triangle) as a function of frequency. (a) Fe^{2+}-12w/v%, (b) Fe^{2+}-15w/v%, (c) Ni^{2+}-12w/v%, (d) Ni^{2+}-15w/v%, (e) Zn^{2+}-12w/v%

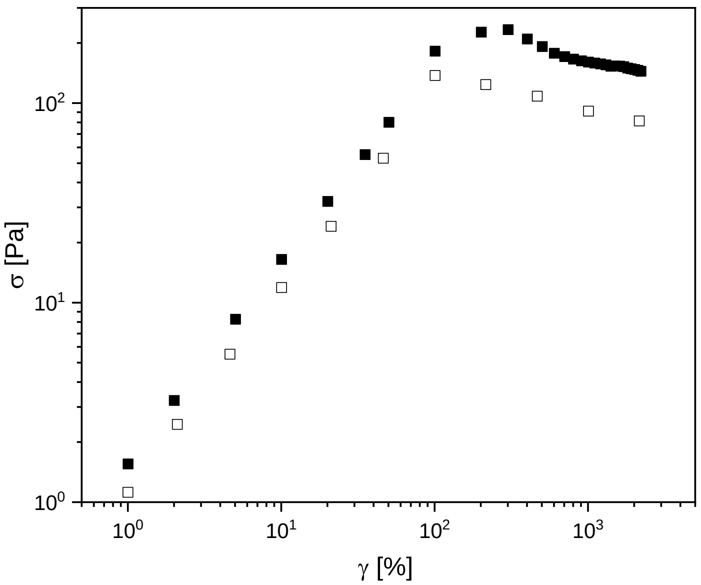

Figure S2. The stress as a function of strain in the oscillatory test at 10 rad/s for Ni^{2+}-12w/v% (solid symbol) and Fe^{2+}-12w/v% (open symbol).
